# Design of Instrumented Shoes for Gait Characterization: A Usability Study With Healthy and Post-stroke Hemiplegic Individuals

**DOI:** 10.3389/fnins.2018.00459

**Published:** 2018-07-20

**Authors:** Dhaval Solanki, Uttama Lahiri

**Affiliations:** Electrical Engineering, Indian Institute of Technology Gandhinagar, Gandhinagar, India

**Keywords:** stroke, gait characterization, gait parameter, instrumented shoe, FSR

## Abstract

Ambulation is a fundamental requirement of human beings for enjoying healthy community life. A neurological disorder such as stroke can significantly affect one's gait thereby restricting one's active community participation. To quantify one's gait, spatiotemporal gait parameters are widely used in clinical context with different tests such as 10 meter walk test, 6 min walk test, etc. Though these conventional observation-based methods are powerful, yet they often suffer from subjectivity, a scarcity of adequately trained therapists and frequent clinical visits for assessment. Researchers have been exploring the technology-assisted solutions for gait characterization. There are laboratory-based stereophotogrammetric methods and walk mats that are powerful tools as far as gait characterization is concerned. However, these suffer from issues with portability, accessibility due to high cost, labor-intensiveness, etc. Faced with these issues, our present research tries to investigate and quantify the gait abnormalities in individuals with neurological disorder by using a portable and cost-effective instrumented shoes (*Shoes*_*FSR*_ *henceforth*). The in-house developed *Shoes*_*FSR*_ comprised of a pair of shoes instrumented with Force Sensing Resistors (FSR) and a wireless data acquisition unit. The real-time FSR data was acquired wirelessly and analyzed by a central console to offer quantitative indices of one's gait. Studies were conducted with 15 healthy participants and 9 post-stroke survivors. The spatiotemporal gait parameters of healthy participants measured using *Shoes*_*FSR*_ were validated with standard methods such as stereophotogrammetric system and paper-based setup. Statistical analysis showed good agreement between the gait parameters measured using *Shoes*_*FSR*_ and the standard methods. Specifically, the mean absolute error of the spatial parameters measured by the *Shoes*_*FSR*_, in the worst case, was 1.24% and that for the temporal parameters was 1.12% with that measured by standard methods for healthy gait. This research shows the potential of the *Shoes*_*FSR*_ to quantify gait abnormality of post-stroke hemiplegic patients. In turn, the results show a promise for the future clinical use of the *Shoes*_*FSR*_.

## Introduction

Reports from World Health Organization suggest that every year ~15 million people suffer from stroke globally (Rodrigo et al., 2013). Among these, ~6 million people die and another 4 million people suffer from partial disability, such as, hemiplegia, that can adversely affect their mobility. Movement disorders associated with such conditions can jeopardize one's independence to perform activities of daily living (ADL). Deficits in balance and gait disorder are common in individuals with hemiplegic stroke. Literature shows that the weight-bearing capacity of the paretic lower limb of a post-stroke survivor can be reduced by up to 43%. Consequently, these patients are often prone to falls with subsequent injuries during over-ground walk (Hagell et al., 1999; Wolfson, 2001). Thus, it is critical to objectively quantify or characterize the residual abilities of the patient before deciding on the therapeutic demands. Researchers have reported that there exists a link between one's gait (defined as a manner of walking) characteristics and different clinical conditions such as, foot inversion, flat foot, etc. that are common after-effects post-stroke (Wang et al., 2009). Again, characterization of one's gait is important since it can be useful in monitoring any improvement in one's gait performance and functional recovery post-stroke due to rehabilitation (Von Schroeder et al., 1995; Alexander, 1996).

Literature shows different approaches, ranging from very simple to extremely complex methods being used for gait characterization. For example, a simple and inexpensive method to characterize one's gait is to record one's footprints during one's over-ground walk (O'Sullivan et al., 2013). In this, one uses paint, ink, or chalk to color the bottom of the patient's foot or shoe so that the foot imprints can be stored post over-ground walk. This is often coupled with the standard walk tests, e.g., 6-min/10-m walk test (O'Sullivan et al., 2013) used to measure one's spatial gait parameters, namely, stride length, step length, etc. (O'Sullivan et al., 2013). To estimate one's temporal gait parameters, e.g., stride time, step time, etc., one often uses a stopwatch during the over-ground walk. Though this approach of characterizing gait is powerful and often practiced in conventional settings, yet this suffers from the subjectivity of measurement, limited healthcare resources, etc. (Toro et al., 2003).

Faced with these challenges, researchers have used different technology-assisted solutions, namely, stereophotogrammetric systems (Cappozzo et al., 2005), walk mats (Muro-De-La-Herran et al., 2014), wearable devices (Wang et al., 2009; Tao et al., 2012), etc. for gait characterization. The stereophotogrammetric systems [such as VICON (VICON Camera System; Vicon Motion Systems Ltd., United Kingdom) motion capture system]^1^ can provide the instantaneous position of markers located on one's body (Cappozzo et al., 2005) that can be used to characterize one's gait. These systems though powerful suffer from large setup time, operational complexity due to the specialized technical knowledge needed to operate the system, high cost, restriction to lab-based settings, etc. (Della Toffola et al., 2012). Researchers have also explored walk mats [such as Gait Mat (Strideway System; Tekscan, Inc., United States)^2^ and GAITRite (GAITRite Classic; CIR Systems, Inc., New Jersey)^3^] These, though costly, are easier to be setup compared to the stereophotogrammetric systems. These walk mats consisting of an array of force switches/sensors can be used to quantify the spatiotemporal gait parameters and also offer dynamic pressure mapping of footprints (O'Sullivan et al., 2013). Though literature reports that the spatiotemporal gait parameters can be reliably measured using walk mats (Barker et al., 2006), yet their usage is limited to well-instrumented laboratories and over-ground gait exercises.

Thus, researchers have explored cost-effective and portable solutions such as wearable sensors to characterize one's gait. The wearable sensors, such as accelerometers, gyro sensors, goniometers, force sensors, etc. can be attached to one's body for characterizing gait (Tao et al., 2012). The Inertial Measurement Unit (IMU) consisting of accelerometers and gyroscopes can be used for gait characterization based on the measurement of position and orientation of the limb as used in different studies (Lemoyne et al., 2008; Yang and Hsu, 2010; Fraccaro et al., 2014). However, the accelerometer and gyroscope-based techniques enable estimation of spatiotemporal gait parameters using model-based approach instead of direct sensor-based measurements (Ladha et al., 2016). Additionally, these need frequent calibration to address drift issues (Tong and Granat, 1999; Alvarez et al., 2008). On the other hand, goniometers can provide angular information but also require mathematical modeling techniques to predict one's spatiotemporal gait parameters (Maranesi et al., 2014) similar to the accelerometer and gyroscope.

Thus, researchers (Huang et al., 2007a; Beauchet et al., 2008; Hanlon and Anderson, 2009) have started exploring the use of portable force sensors such as, force sensitive resistors (FSRs). Using FSRs fitted below one's feet, we can detect one's gait-related events such as heel strike, toe off, etc. that in turn can be used to measure spatiotemporal gait parameters. There is a rich literature that speaks on the use of FSR. For example, different researchers have used multiple FSRs ranging from 2 to 32 numbers located at different positions under one's feet, namely, toe, heel, ball metatarsal 1 to 5, etc. (Chen et al., 2007, 2008; Huang et al., 2007b; Pawin et al., 2011; Pinkam and Nilkhamhang, 2013; Majumder et al., 2015) to characterize one's gait. Usage of multiple FSRs, though lend improved precision of measurements, yet, it increases the hardware complexity by increasing the number of data channels to be handled along with difficulty in troubleshooting, locating and correcting faulty FSR(s), making it infeasible for practical applications. On the other hand, too few FSRs can miss picking up certain aspects of gait abnormalities, such as foot inversion/eversion (Perry and Lafortune, 1995) often seen in post-stroke patients. All of the FSR-based applications have used one heel sensor along with other sensors situated at different locations under the feet. Instead of using one FSR sensor at the center of the Heel, we placed two FSRs at the heel location spaced at ~30 mm apart to accommodate patients having foot inversion/eversion. Having already used two FSR sensors, we wanted to keep the sensing circuit less complex and thus we kept a minimum of one more FSR sensor at the toe with which we were able to extract a number of spatiotemporal features that could characterize even pathologic gait. Most of the currently existing FSR-based studies characterizing one's gait have been applied to participants demonstrating healthy gait. Thus, further exploration to carry out detailed gait characterization of unhealthy gait such as for post-stroke hemiplegic patients using FSR-based approach is warranted.

Motivated by this need, in our present research, we have developed a pair of shoes instrumented with FSRs. It features two heel sensors and one toe sensor. The *Shoes*_*FSR*_ features wireless data acquisition module that allows continuous and unobstructive assessment of gait features even outside the laboratory environment. The *Shoes*_*FSR*_ can be used to detect different gait-related events such as heel strike, heel off, toe strike and toe off, useful for extracting different gait parameters. In order to obtain a faithful representation of the gait characteristics, it is essential that the gait parameters be validated with those measured using standard techniques. This is critical, since, literature reports trade-off between the accuracy and portability of gait measuring systems and their clinical usage (Beauchet et al., 2008). In fact, though, few studies such as by Lopez-Meyer et al. (2011) using FSR-based shoes for post-stroke individuals showed promising results as far as extraction of limited gait parameters (only temporal) are concerned, yet, the researchers did not report on validation of their shoes with any standard stereophotogrammetric techniques.

While our *Shoes*_*FSR*_ could measure one's spatiotemporal gait parameters during the over-ground walk, we validated our observations using standard setups, namely, VICON and paper-based setups. The objectives of our study were three-fold, namely, (i) Design a pair of FSR-based shoes (*Shoes*_*FSR*_) that can measure spatiotemporal gait parameters for both healthy and post-stroke hemiplegic participants, (ii) Validate the temporal and spatial gait parameters of healthy participants using VICON and paper-based setup, respectively, during one's over-ground walk and (iii) Use *Shoes*_*FSR*_ to quantify and paper-based setup to validate the spatial gait parameters of post-stroke hemiplegic participants.

This paper is organized as follows: section Materials and Methods presents the system design followed by the methodology used for the study. Section Result and Discussion offers the results obtained during the study. Finally, section Discussion and Limitation summarizes the research findings and discusses the limitations of the current research as well as the direction of future research.

## Materials and methods

### Gait characterization system

Our system comprised of four sub-modules, such as (A) Instrumented Shoes with Force Sensitive Resistors (FSR) (*Shoes*_*FSR*_ henceforth) (B) Data Acquisition (C) VICON-*Shoes*_*FSR*_ synchronizer and (D) Feature extraction modules. The data collection process was carried out in three stages, namely, Stage 1, Stage 2, and Stage 3. Stage 1 was used to validate the temporal gait parameters measured by *Shoes*_*FSR*_ with that by VICON. This used all the modules [modules (A) to (D)]. Stage 2 was used to validate the spatial gait parameters measured by the *Shoes*_*FSR*_ with that by the paper-based setup. This incorporated modules (A), (B), and (D). The Stages 1 and 2 were carried out with healthy participants. Stage 3 was similar to Stage 2, except that it was used to collect experimental data with post-stroke hemiplegic participants.

#### Instrumented shoes with FSR *(shoes_*FSR*_)*

**Figure 1:**
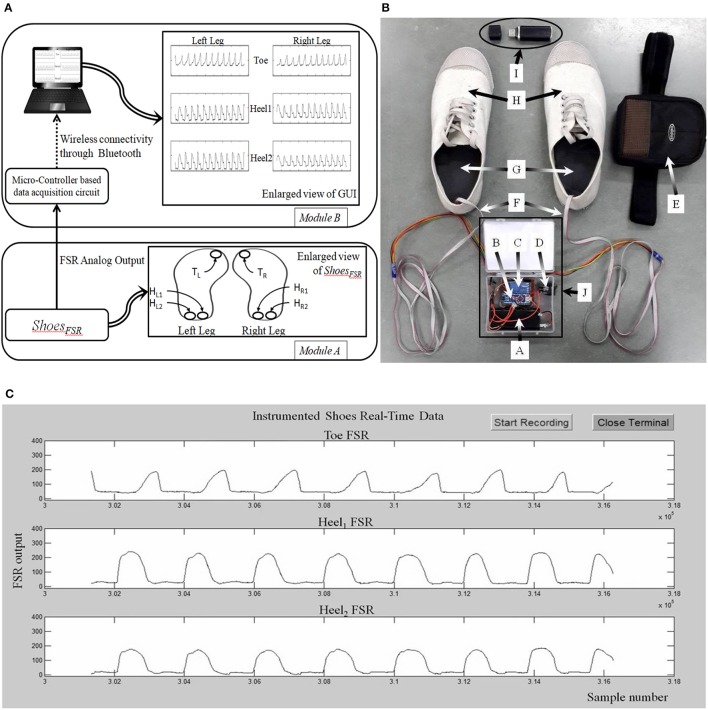
**(A)** Block Diagram of *Shoes*_*FSR*_; **(B)** Photo of *Shoes*_*FSR*_ (Physical appearance) with Data Acquisition Module (DAQ); **(C)** A snapshot of graphical user interface. T_L_/T_R_: FSR_GD_ placed below Left and Right toe respectively; H_L1_/H_R1_: 1st FSR_GD_ placed below Left and Right heel; H_L2_/H_R2_: 2nd FSR_GD_ placed below Left and Right heel; A: Battery for DAQ; B: Micro-controller circuit for DAQ; C: Bluetooth Transmitter; D: ON/OFF switch for DAQ; E: Case to mount DAQ at the subject's pelvis; F: Wires connecting DAQ and FSR_GD_ mounted in shoe-insole; G: Shoe-insole mounted with FSRs; H: *Shoes*_*FSR*_; I: Bluetooth Receiver; J: Data Acquisition Module.

Figure 1 shows the block diagram of the data acquisition system using the *Shoes*_*FSR*_ as one of the modules. The *Shoes*_*FSR*_ comprised of a pair of shoes having six FSRs (three FSRs in each shoe for characterizing the Gait Dynamics (FSR_GD_ *henceforth*) placed below the shoe insole. To avoid physical damage to the FSRs due to the forces exerted during one's walk, an extra protective layer of dummy insole was placed above the shoe insole mounted with FSRs. One of the common faults that can occur is the breakage of the electrical connections. However, we provided heat shrinks to offer insulation and strengthening of electrical connections between wires and the terminals of FSRs. Insole mounted with FSRs was crafted with grooves to conceal signal carrying wires in the insole protecting the wires from unwanted external forces during one's walk. Six FSR_GD_ (T_L_, H_L1_, H_L2_ for left shoe and T_R_, H_R1_, H_R2_ for right shoe) were placed below the shoe insole at different positions below the foot, namely, Toe and two Heel locations [Figure 1A, Module (A)]. The idea of placing two FSRs at the Heel of each shoe was to use the *Shoes*_*FSR*_ to measure one's gait parameters (such as stride time, step time, stance time, swing time, etc.) for characterizing both healthy and pathologic gait. Specifically, post-stroke patients often demonstrate pathologic gait (Perry and Lafortune, 1995). For example, the pathologic gait commonly seen in post-stroke survivors is often accompanied with foot inversion causing abnormal weight shifting during over-ground walk (Chae, 2016).

#### Data acquisition module

Data from the *Shoes*_*FSR*_ was in the form of 0–5 V analog signal. The analog signal from 6 Nos. of FSRs in the pair of shoes were routed to 6 analog input pins of the ATMEGA328 (microcontroller) that was powered with a regulated 5 V battery supply. This data was processed by the microcontroller of the Data Acquisition Module (that was pre-programmed using an Interactive Development Environment that comes with this microcontroller) [Figure 1A (Module B) and Figure 1B]. The data was transmitted to a remote laptop using a pair of serial Bluetooth device (HC-05). The sensor data was sampled using a 10-bit analog to digital convertor (ADC) at ~200 samples/second that was transmitted to the remote data logger computer (laptop) at a baud rate of 115.2 Kbits/second. This data was presented on a Graphical User Interface (GUI) (Figure 1C) in real-time. The experimenter used the GUI to confirm the health of the FSR sensors.

#### VICON-*shoes_*FSR*_* synchronizer

**Figure 2:**
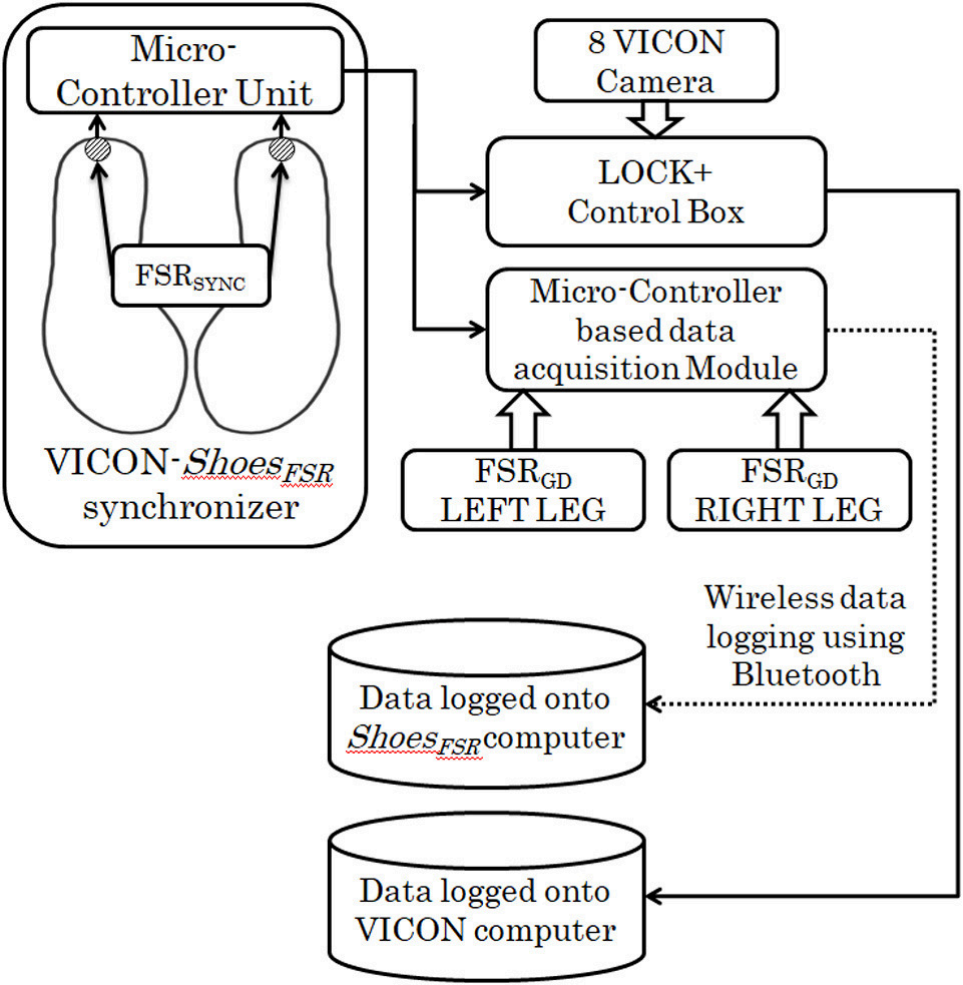
Block Schematic of VICON-*Shoes*_*FSR*_ synchronizer.

This study was conducted in three different stages. In Stage1 of the study, VICON was used to validate the temporal gait parameters measured by *Shoes*_*FSR*_. For the validation study, it was essential to synchronize the VICON and *Shoes*_*FSR*_. Therefore, we used the data synchronizing module (VICON-*Shoes*_*FSR*_ synchronizer) that provided a simultaneous synchronizing marker to both systems (i.e., VICON and *Shoes*_*FSR*_). Figure 2 shows the block schematic of the setup used for VICON-*Shoes*_*FSR*_ synchronization module. The data synchronizing module consisted of a microcontroller-based unit that received marker input from synchronizing FSR (FSR_SYNC_) mounted at the heel of the *Shoes*_*FSR*_ (one to each of the two shoes; Figure 2). Thus, there were eight inputs from the *Shoes*_*FSR*_ (six from the FSR_GD_ and two from FSR_SYNC_)_._ Here we wanted to use the FSR_SYNC_ to send binary (ON/OFF; ON indicating heel contact; OFF indicating no heel contact) marker signal to the synchronization module. The Synchronizing marker signal based on No Contact/Heel Contact was transmitted simultaneously to (i) Data Logger Computer of VICON system via Lock+ hardware module and (ii) Data Logger Computer of *Shoes*_*FSR*_ via the Microcontroller based Data Acquisition Circuit (Figure 2). The Lock+ hardware module (Lock+ Sync Box; Vicon Motion Systems Ltd, United Kingdom)^4^ that came with VICON was used to connect and synchronize external inputs e.g., from FSR_SYNC_.

#### Feature extraction module

**Figure 3:**
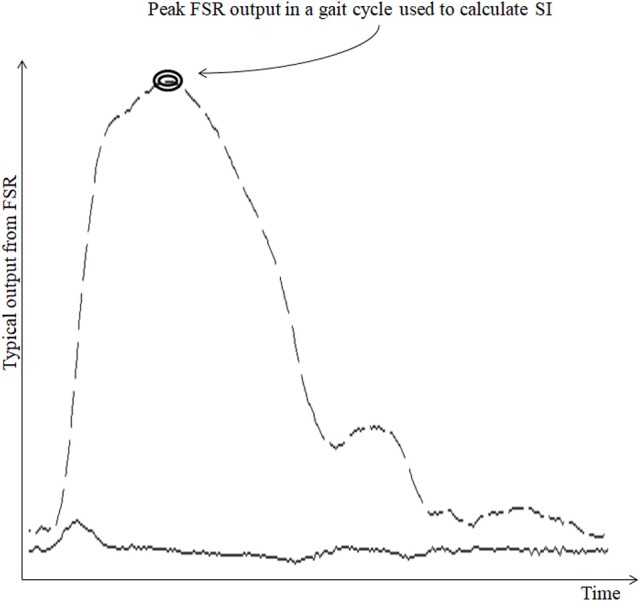
Typical output from FSR sensor. Broken line shows the typical output from FSR_GD_ sensor for one gait cycle; Solid line (lower continuous line) shows the computed successive difference in FSR output; “SI” stands for Symmetry Index.

The raw data (Figure 3) from the *Shoes*_*FSR*_ were acquired by the Data Acquisition Module and stored in the Data Logger Computer for offline analysis. This raw data was filtered by a 10 point (window of ~50 ms) moving average filter thereby achieving a Signal:Noise Ratio (SNR) of ~40 dB. The window length of ~50 ms was chosen as an initial approximation. The filtered data was processed to extract relevant features such as stride length, stride time, etc. Additionally, a video of participant's over-ground walk was recorded for subsequent offline analysis.

##### Computation of stride time (*GF*_*1*_)

**Figure 4:**
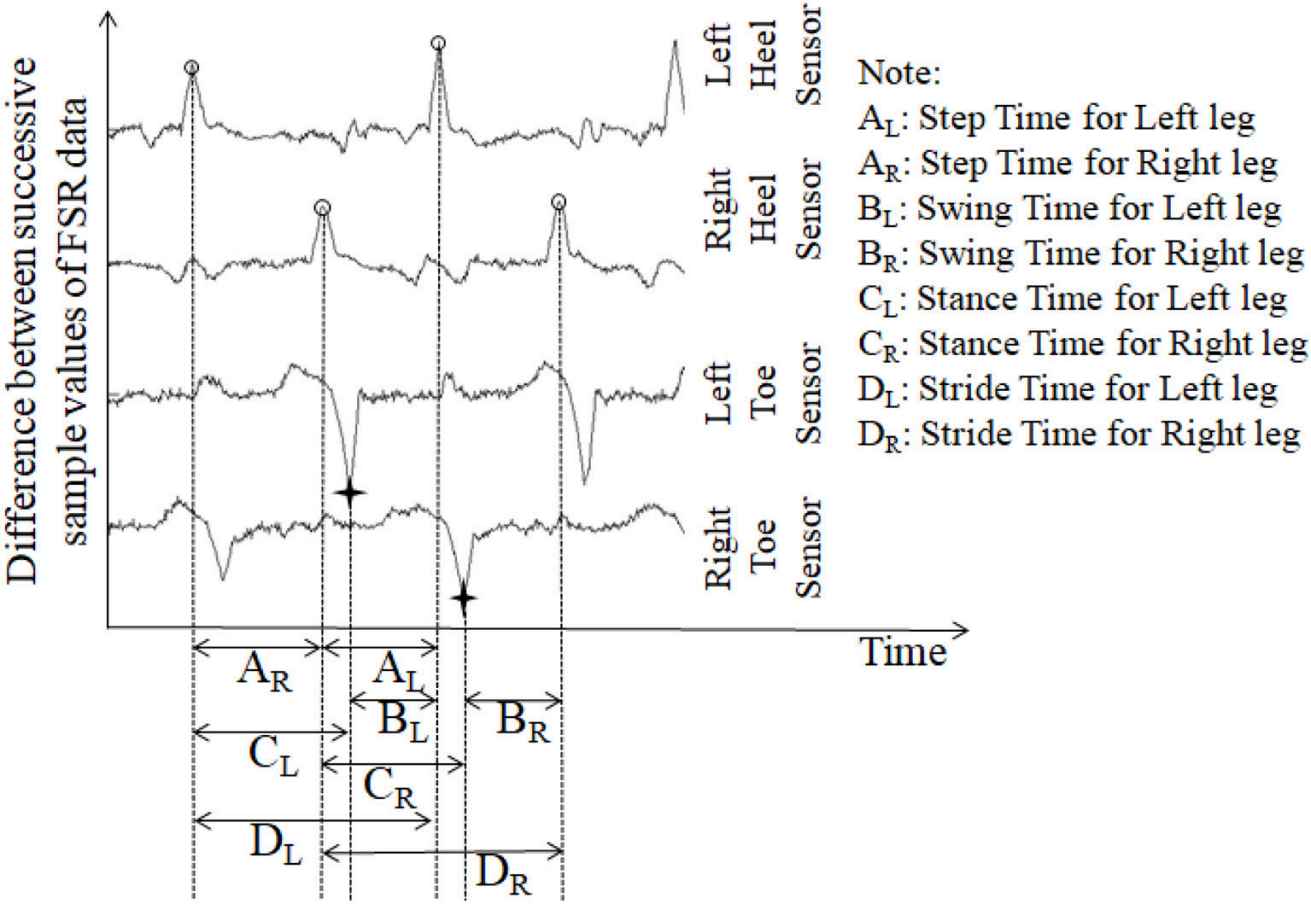
The successive difference signal derived from different FSR outputs. “o” represents heel strike (foot strike) events; “+” represents toe off (foot off) event.

Stride time can be calculated as the time difference between two successive heel strikes of the same foot (O'Sullivan et al., 2013). While a participant walked wearing the *Shoes*_*FSR*_, the microcontroller based wireless Data Acquisition Module acquired and transmitted the FSR_GD_ data (Figure 3 shows a typical example of filtered data) to the remote Data Logger Computer (laptop). The filtered data from the heel sensors (FSR_GD_) was processed to compute the difference between successive sample values (V_*n*_ − V_n−1_; where “V” is the digital value (ranging from 0 to 1023) obtained at the output of the 10 bit ADC; “*n*” is the sample number) of the data (Figures 3, 4). As can be seen from Figure 4, the peak points were picked up automatically by our in-house developed algorithm from the successive difference of values of consecutive samples of heel FSR_GD_ (as shown by lower solid line in Figure 3) to extract heel strike events. Similarly, the toe off events were extracted from the trough points of the successive difference signal computed from signal recorded by the toe FSR_GD_. The Figure 4 represents the time-stamped heel-strike and toe-off events indicated by “o” and “+,” respectively for both the legs. Subsequently, the Stride Time was calculated from the time interval between two successive heel-strike events [represented by “D_L_” and “D_R_” in Figure 4 for each of Left and Right legs (Leg_L_ and Leg_R_ respectively, *henceforth*), respectively].

##### Computation of stride length (*GF*_2_)

Stride Length is the distance covered between two successive heel strikes of the same foot (O'Sullivan et al., 2013). To compute the Stride Length, we needed information on Stride Time [section Computation of Stride Time (*GF*_1_)] and Walking Speed. To measure one's Walking Speed, we used the recorded video of the participant's walk that provided information on the time taken by an individual to cover a pre-defined walk distance (specified by the experimental setup; section Experimental Setup below). While a participant walked wearing the *Shoes*_*FSR*_, the Stride Time was multiplied with Walking Speed to compute the individualized Stride Length (Frenkel-Toledo et al., 2005) for each of Leg_L_ and Leg_R_. Subsequently, the Normalized Stride Length (Stride Length_Norm_) was computed based on one's height (Equation 1) to nullify the effect of inter-subject height differences that can affect one's gait parameters (Elble et al., 1991).

(1) $$Stride\;Length_{Norm}=\frac{Walking\;Speed\;*\;Stride\;Time}{Height}$$

##### Computation of step time (*GF*_3_)

The *Shoes*_*FSR*_ data was collected and logged on the remote Data Logger Computer for offline processing. One's Step Time can be calculated as the time interval between two successive heel-strike events of contralateral legs (O'Sullivan et al., 2013). Here, we used our logged data to calculate the Step Time from the time interval between two successive heel-strike events of contralateral legs (represented by “A_R_” and “A_L_;” Figure 4).

##### Computation of step length (*GF*_4_)

One's Step Length can be computed from the spatial distance between two successive heel-strike events of contralateral legs (O'Sullivan et al., 2013). Thus, this depends on one's Step Time [section Computation of Step Time (*GF*_3_)] and Walking speed. We computed the Step Length from the product of the Step Time and Walking speed (Frenkel-Toledo et al., 2005) for each leg. Again, to nullify the effect of inter-subject height differences, we computed the normalized Step Length (Step Length_Norm_) using Equation (2).

(2) $$Step\;Length_{Norm}=\frac{Walking\;Speed\;*\;Step\;Time}{Height}\;(2)$$

##### Computation of single support time (*GF*_5_)

Single (limb) Support Time of a gait cycle is the duration for which only one leg supports the body during one's gait (Debi et al., 2011). Alternatively, Single Support Time for one leg (say, left) can be measured from the Swing Time for the other leg (say, right) (Bagley et al., 1991). Here, we considered an alternate approach to measure the Single Support Time. Subsequently, the % Single Support Time for each leg (Leg_L_ and Leg_R_) was calculated as a percentage of the total gait cycle time using Equations (3, 4), respectively (Figure 4).

(3) $$\begin{array}{l} \%\;Single\;Support\;Time_{L}\;=\;\frac{Swing\;Time(B_{R})}{Gait\;Cycle\;Time(D_{L})}*\;100\% \end{array}$$

(4) $$\%\;Single\;Support\;Time_{L}\;=\;\frac{Swing\;Time(B_{L})}{Gait\;Cycle\;Time(D_{R})}*\;100\%$$

##### Computation of parameters (*GF*_6_) related to swing and stance phases of gait

One's gait cycle can be divided into two phases, such as, Stance and Swing phases that have been reported to be ~60 and 40% of one's gait cycle for healthy gait (O'Sullivan et al., 2013). The Stance and Swing phases (associated with a reference foot) is related to the foot being in contact and not in contact with the ground surface, respectively (O'Sullivan et al., 2013). In our study, the gait cycle time (Hausdorff et al., 1998) was calculated as the time interval between two successive heel-strike events of the same leg [represented by “D_L_” and “D_R_” in Figure 4 as discussed in section Computation of Stride Time (*GF*_1_)]. From this, we computed the Stance and Swing phases as percentage of the gait cycle. Specifically, we used the period of the Stance and Swing phases to calculate the percentage of the gait cycle time used by each reference leg. For this, the data from the heel sensors and the toe sensors (FSR_GD_) of *Shoes*_*FSR*_ were processed to extract information on the time-stamped heel-strike and toe-off events (indicated by “o” and “+” for heel-strike and toe-off events, respectively for both the legs; Figure 4). Subsequently, the Swing Time was calculated as the time interval between successive toe-off and heel-strike events of the same foot when the foot is not in contact with the ground (represented by “B_L_” and “B_R_” in Figure 4). Similarly, the Stance Time was calculated when the foot is in contact with the ground surface (represented by “C_L_” and “C_R_” in Figure 4). The % Swing (*GF*_6_*Swing*_) and % Stance (*GF*_6_*Stance*_) Phases were calculated using the Swing Time and Stance Time, respectively and quantified as a percentage of the total gait cycle time of the reference leg (Equations 5–8).

(5) $$\%\;Swing\;Phase_{L}=\frac{Swing\;Time(B_{L})}{Gait\;Cycle\;Time(D_{L})}*\;100\%$$

(6) $$\%\;Stance\;Phase_{L}=\frac{Stance\;Time(C_{L})}{Gait\;Cycle\;Time(D_{L})}*\;100\%$$

(7) $$\%\;Swing\;Phase_{R}=\frac{Swing\;Time(B_{R})}{Gait\;Cycle\;Time(D_{R})}*\;100\%$$

(8) $$\%\;Stance\;Phase_{R}=\frac{Stance\;Time(C_{R})}{Gait\;Cycle\;Time(D_{R})}*\;100\%$$

##### Computation of symmetry index (SI) (*GF*_7_)

Since we had hemiplegic post-stroke patients with gait abnormalities who volunteered in our study, we wanted to quantify the contribution of each leg toward the overall gait in an individualized manner. For this, in Stage 3 of this study, we computed the Symmetry Index (SI). Literature indicates the use of SI, based on gait parameters such as Step Length, Step Time, Stance Time, Swing Time, etc. (Błazkiewicz et al., 2014). Again, other studies report that the post-stroke hemiplegic patients often suffer from assymetric weight-bearing capacity on both sides of the body. For example, the weight-bearing capacity of the paretic lower limb of a post-stroke survivor can be reduced by up to 43% (Hagell et al., 1999; Wolfson, 2001). Thus, considering the individualized gait profile and the weight-bearing capacity, we wanted to select at least one of the heel sensors and explore the force profile measured by the two heel sensors (FSR_GD_ at the heel) for each participant while computing the SI. Depending on one's gait profile such as healthy gait or pathologic gait (e.g., with foot inversion/eversion, etc.), our system selected the analog output from at least one of the two FSR_GD_ at the heel based on whichever sensor first indicated heel strike with the ground surface during gait. As regards the force profile measured by the two heel sensors, instead of using the maximum of the peak values of the force profile recorded by the two FSR_GD_ at the heel (of each leg), we considered the minimum of the peak (magnitude) values (Minimum Peak Value, MPV) of the data for each leg. Specifically, the MPV was chosen as representative of the worst case magnitude of the force profile since we wanted to investigate the maximum possible asymmetry in one's gait. For example, if an individual has foot inversion, it might so happen that the force profiles measured by the two FSR_GD_ at the heel of each leg are quite different. In such cases, the MPV can characterize the worst-case asymmetry in one's gait. Using MPV, we computed the SI value using Equation (9).

(9) $$SI=\frac{\left|Xl-Xr\right|}{0.5(Xl+Xr)}*100\%$$

Where *Xl* and *Xr* are the MPV values corresponding to the Leg_L_ and Leg_R_, respectively.

##### Tests of correlation and statistical significance

In this study, we computed different gait parameters (*GF*_1_ to *GF*_7_) from the data measured by the *Shoes*_*FSR*_. In Stage 1 and Stage 2 of this study, we wanted to validate the ability of *Shoes*_*FSR*_ to measure different gait parameters by using the state-of-the-art (standard) methods such as VICON and paper-based setup. For this, we calculated the Intra-class Correlation Coefficient (ICC) (Shrout and Fleiss, 1979) to test the conformity of gait parameters obtained using *Shoes*_*FSR*_ with those computed by VICON and paper-based setup. In Stage 3 of this study, we wanted to understand the implications of stroke on one's gait parameters. While analyzing our results (i.e., gait parameters) of Leg_L_ and Leg_R_ of each participant, we wanted to understand whether there existed any statistically significant difference in gait parameters obtained from both the legs of stroke patients. Considering limited sample size, we applied nonparametric Wilcoxon–Mann–Whitney test (Field, 2013) to identify any statistically significant difference.

### Participants

Here we conducted studies in three Stages, namely, Stage 1, Stage 2 and Stage 3. Table 1 shows the participants' characteristics for Stages 1 and 2. In Stage 1, five male healthy participants [Mean (SD) = 27 (±3.67) years and Body Mass Index (BMI) of Mean (SD) = 21.98 (±2.33) kg/m^2^] volunteered. In Stage 2, ten male healthy participants (Mean (SD) = 25.8 (±3.11) years, BMI of Mean (SD) = 22.21 (±2.33) kg/m^2^) volunteered. The healthy participants were recruited from the neighborhood. In Stage 3, nine male hemiplegic post-stroke patients [Mean (SD) = 46 (±10.44) years, and BMI of Mean (SD) = 23.8 (±2.89) kg/m^2^] volunteered. The post-stroke participants (Table 2) were recruited from nearby physiotherapy hospital where they were undergoing treatment. Enrolment of these participants was through physiotherapist's referral.

**Table 1:** Participant characteristics for Stages 1 and 2.

**Stage ID**	**Participant ID**	**Age in Range (Year)**	**Height (cm)**	**BMI(kg/m^2^)**
Stage 1	P1	21–25	167	24.2
	P2	25–30	175	24.4
	P3	31–35	165	19.9
	P4	25–30	171	19.4
	P5	21–25	164	22
Stage 2	P6	21–25	167	24.2
	P7	21–25	178	20.4
	P8	25–30	175	24.4
	P9	31–35	165	19.9
	P10	25–30	171	19.4
	P11	25–30	175	21.7
	P12	25–30	171	22.2
	P13	21–25	166	22.7
	P14	25–30	170	22.9
	P15	21–25	179	24.3

*BMI, Body Mass Index; Stage 1: Validation of gait parameters measured using Shoes_FSR_ with those measured using VICON setup; Stage 2: Validation of gait parameters measured using Shoes_FSR_ with those measured using paper-based setup*.

**Table 2:** Participant characteristics for Stage 3 of study.

**Participant ID**	**Age in range (Year)**	**Height (cm)**	**BMI (kg/m^2^)**	**Hemiplegic side (Right/Left)**	**Post-stroke period (Months)**	**Time taken to complete 10 MWT (s)**
S1	25–30	168	26.6	Right	12	16.65
S2	56–60	151.5	23.6	Right	42	11.09
S3	56–60	166.5	24	Left	36	56
S4	41–45	168	22.4	Left	12	7.1
S5	41–45	165	23.5	Right	4	7.54
S6	41–45	166	22.1	Left	1	18.22
S7	56–60	159.5	18.7	Right	48	18.3
S8	41–45	165.5	24.2	Left	24	35.41
S9	35–40	173	29.1	Right	36	25.8

*BMI, Body Mass Index; MWT, Meter Walk Test*.

### Experimental setup

This study was conducted in three Stages having two experimental setups. For example, Stage 1 consisted of *Shoes*_*FSR*_ and VICON and Stages 2 and 3 consisted of *Shoes*_*FSR*_ and paper-based setup.

#### Experimental setup for stage 1

**Figure 5:**
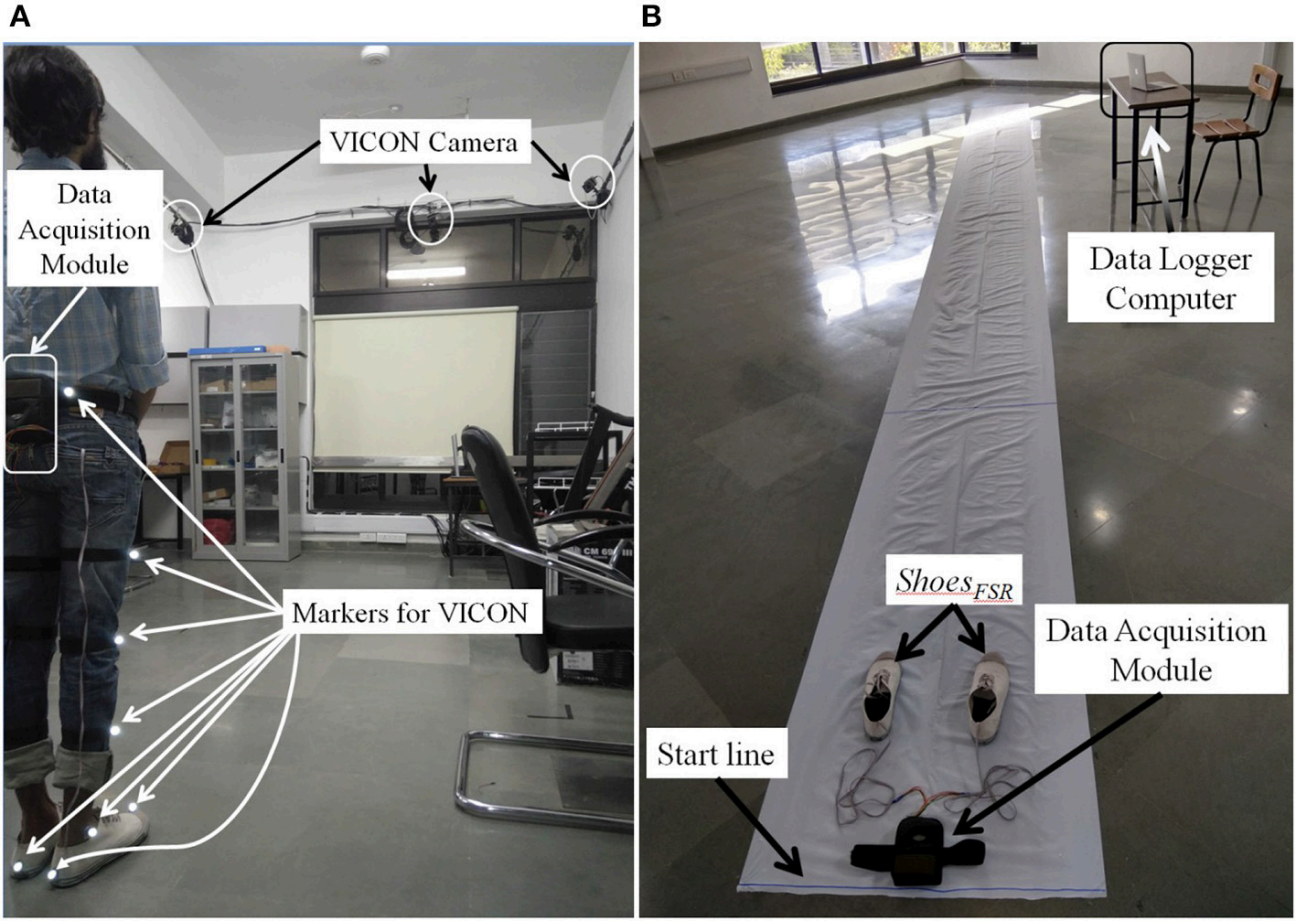
Walk path **(A)** with VICON cameras for Stage 1; **(B)** for Stages 2 and 3.

In Stage 1, we used a 7 m long walk path inside a lab-based setting (Figure 5A) for over-ground walk. This setting was equipped with VICON system that included (i) 8 cameras (2,048 × 1,088 pixels) to track reflective markers placed on the subject, (ii) a LOCK+ hardware module to collect data and integrate the VICON cameras and *Shoes*_*FSR*_ (aided by VICON-*Shoes*_*FSR*_ synchronizer; section VICON-*Shoes*_*FSR*_ Synchronizer) for synchronization and (iii) a computer [Intel(R) Xeon(R) CPU operating at clock frequency of 2.1 GHz with 16 GB RAM and 64-bit windows 10 operating system] to record stereophotogrammetric data (from VICON cameras). A Data Logger laptop was used for storing the gait-related data captured by *Shoes*_*FSR*_ (section Data Acquisition Module) wirelessly. A MATLAB-based Data Acquisition Module (Figure 1C) running on the laptop was used for recording and displaying instantaneous data captured by the *Shoes*_*FSR*_. While the participants walked over-ground on the walk path, data from both the stereophotogrammetric setup and *Shoes*_*FSR*_ were simultaneously recorded during the intermediate 3 m path (that was in the Field of View of the VICON cameras) for measurement and validation of temporal gait parameters. The central 3 m path was chosen as an initial approximation to account for the acceleration and deceleration in one's walk toward the beginning and the end of the path (Henriksen et al., 2004). For measurement and validation of spatial gait parameters, instead of using a limited walk path of length 3 m (that was available to us), we wanted a longer walk path with a possibility of having a fairly constant average walking speed (devoid of acceleration and deceleration) and thereby moved to a paper-based setup that can offer a 10 m walk path and this formed the experimental setup for Stages 2 and 3.

#### Experimental setup for stages 2 and 3

The experimental setup in Stages 2 and 3 comprised of a 10 × 0.7 m walk path (Figure 5B). This walk path was created by pasting a 10 × 0.7 m white paper on the floor. Additionally, the shoe soles of the two *Shoes*_*FSR*_ were colored so to aid the experimenter to make the measurements from the imprints on the white paper of the walk path. While taking the measurements, care was taken that approximately the intermediate 6 m path was considered for the measurement (Henriksen et al., 2004). This was used to account for the effect of one's possible acceleration and deceleration toward the beginning and end of the walk, similar to that during Stage 1.

### Procedure

#### Procedure followed during stage 1

The Stage 1 required a commitment of ~30 min from each participant. The study began with a brief introduction of the experimental setup comprising of *Shoes*_*FSR*_, VICON system along with reflective markers, a 7 m long straight walk path, etc. Then the experimenter told the participant that he would be expected to walk on the straight path at his comfortable speed. Also, the participant was told that he can discontinue from the study at any point of time if he felt uncomfortable. Subsequently, the experimenter helped the participant to wear the *Shoes*_*FSR*_. The reflective markers of VICON system were placed on both the lower limbs of the participant as described in Table 3. Before starting the study, the experimenter confirmed that the participant had understood what he was expected to do and waited for his yes nod to start the study. First, the participant was asked to stand with both legs touching the Start line (Figure 5A). To facilitate synchronization of data (from VICON and *Shoes*_*FSR*_), the participant was asked to make three taps on the ground using the right leg. Then the participants walked on the straight path at their comfortable speed till they reached the end of the path followed by standing upright for ~1–2 s while the experimenter stopped the data collection process.

**Table 3:** Marker placement for VICON system.

**Marker ID**	**Marker Position**
M1	Anterior superior iliac
M2	Posterior superior iliac
M3	Thigh
M4	Knee
M5	Tibia
M6	Ankle
M7	Toe
M8	Heel

#### Procedure followed during stages 2 and 3

Each of the Stages 2 and 3 required ~20 min from each participant. Similar to that in Stage 1, in Stages 2 and 3, the participant was briefed on the experimental setup and was told what he was expected to do during the study. The Stage 2 had healthy participants. The Stage 3 involved post-stroke hemiplegic patients. The patients were recruited through referrals and a physiotherapist in our team checked the inclusion criteria. In this, the patients were checked for their ability to perform the 10 m walk-test (O'Sullivan et al., 2013) while walking over-ground without any external support such as orthosis, canes, etc.

Subsequently, the experimenter helped the participant to wear the *Shoes*_*FSR*_ with the bottom of the shoe sole being colored. The participant was asked to stand with both the legs touching the Start line (Figure 5B). A video of participant's walk was recorded for post analysis. In order to synchronize data from *Shoes*_*FSR*_ and video, the participant was asked to make three taps on the ground using the right leg before starting to walk. The participants walked at their comfortable speed till they reached the end of the path followed by standing upright for ~1–2 s while the experimenter stopped the data collection process.

## Result and discussion

In our study, participants were asked to perform over-ground walk while wearing *Shoes*_*FSR*_. The results of all the three stages along with the post-study feedback are discussed below.

### Post-study feedback

After the participants wearing *Shoes*_*FSR*_ had finished their walk on the 10 m walk path, a questionnaire was used by the experimenter to get the participants' feedback on our system. We were interested to know their views on whether they (i) felt any inconvenience in wearing the *Shoes*_*FSR*_, (ii) faced any difficulty in understanding the task, (iii) agreed to use the *Shoes*_*FSR*_ again and (iv) are willing to refer others to participate in our study. From the participants' responses, we found that the participants did not experience any inconvenience while wearing the *Shoes*_*FSR*_. Also, they did not report any difficulty in understanding the tasks. Additionally, they expressed their willingness to use *Shoes*_*FSR*_ in future and also refer their known acquaintances to this study. Thus, from the participants' feedback, we can infer that the *Shoes*_*FSR*_ has the potential to be accepted by individuals with gait disorder.

### Results on validation of temporal gait parameters computed using the *shoes_*FSR*_* for healthy participants

**Figure 6:**
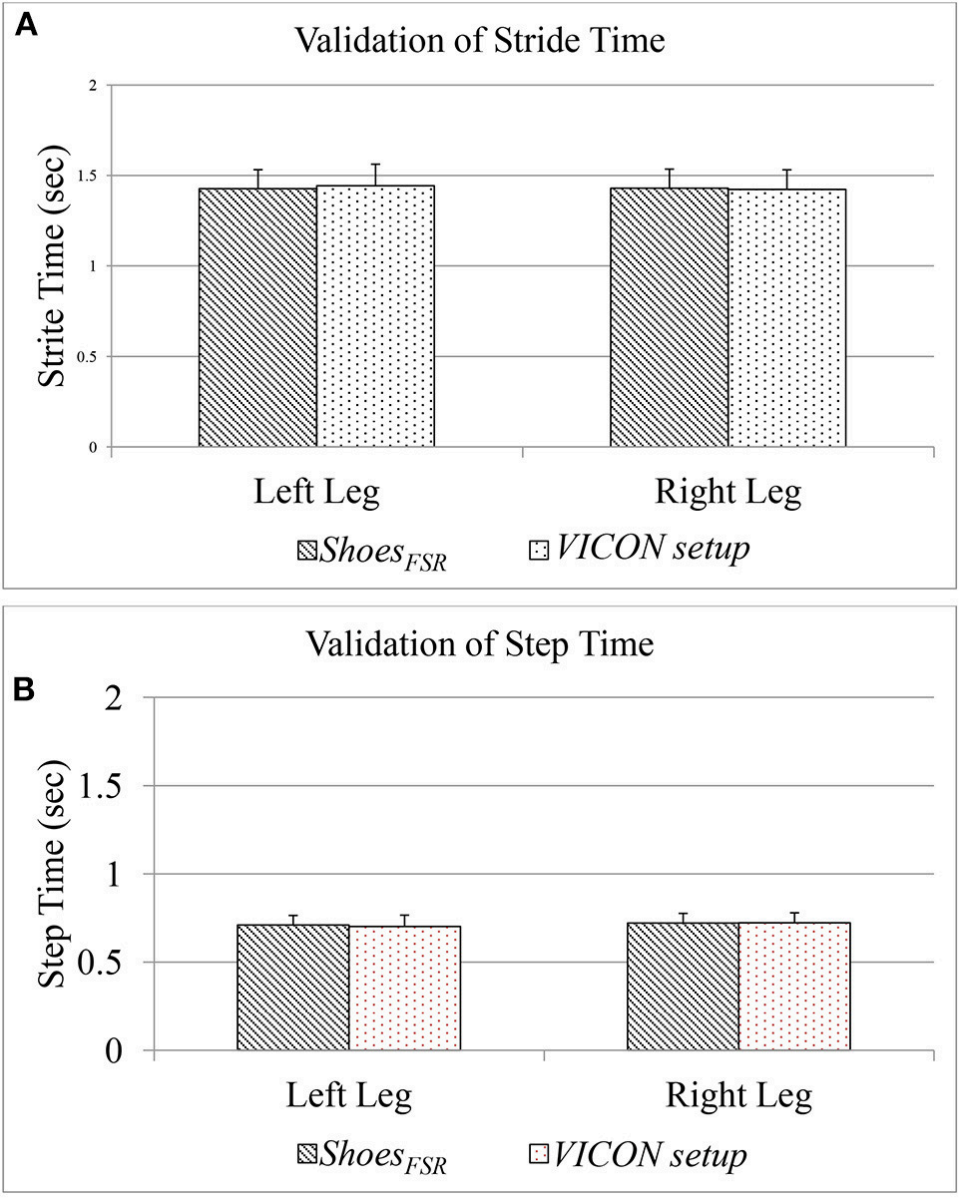
**(A)** Comparison of Stride Time measured using *Shoes*_*FSR*_ and VICON setup; **(B)** Comparison of Step Time measured using *Shoes*_*FSR*_ and VICON setup.

The Figure 6 shows a comparative group analysis of two temporal gait parameters, such as Stride Time (*GF*_1_) and Step Time (*GF*_3_) [for both the left leg (Leg_L_) and right leg (Leg_R_)] measured using the two systems, namely, *Shoes*_*FSR*_ and VICON. The measured values show good agreement between the results (the average % absolute error being 0.70 and 0.72%, respectively for Leg_L_ and Leg_R_ in case of *GF*_1_; 1.12 and 0.48%, respectively for Leg_L_ and Leg_R_ in case of *GF*_3_) computed using the two systems. Additionally, we found a high correlation in terms of Intra-class correlation coefficients (ICC) for *GF*_1_ (ICC = 0.99 and 0.97 for Leg_L_ and Leg_R_, respectively) and *GF*_3_ (ICC = 0.99 and 0.95 for Leg_L_ and Leg_R_, respectively) measured using the *Shoes*_*FSR*_ and VICON. From these results, we can infer that the *Shoes*_*FSR*_ can offer a reliable measure of at least some of the temporal gait parameters.

Please note that, since our participants were healthy individuals, we could observe a close agreement in the gait parameters for Leg_L_ and Leg_R_. Specifically, the difference (%Δ) in the group average of *GF*_1_ and *GF*_3_ between the Leg_L_ and Leg_R_, as measured by *Shoes*_*FSR*_ were ~0.21 and 1.50%, respectively. Also, statistical analysis using non-parametric Wilcoxon–Mann–Whitney test showed no statistical significance (*p* > 0.05) between the measured gait parameters of Leg_L_ and Leg_R._

### Results on validation of spatial gait parameters computed using the *shoes_*FSR*_* for healthy participants

During Stage 2, while the participants walked over-ground with the paper-based setup wearing the *Shoes*_*FSR*_ having colored bottom, the foot imprints were used for subsequent analysis. This was later used to measure normalized spatial gait parameters such as normalized Stride Length (*GF*_2_) and normalized Step Length (*GF*_4_) [discussed in section Computation of Stride Length (*GF*_2_) and Computation of Step Length (*GF*_4_), respectively]. The accompanying video of the participant's walk was analyzed offline to validate the *GF*_2_ and *GF*_4_.

**Figure 7:**
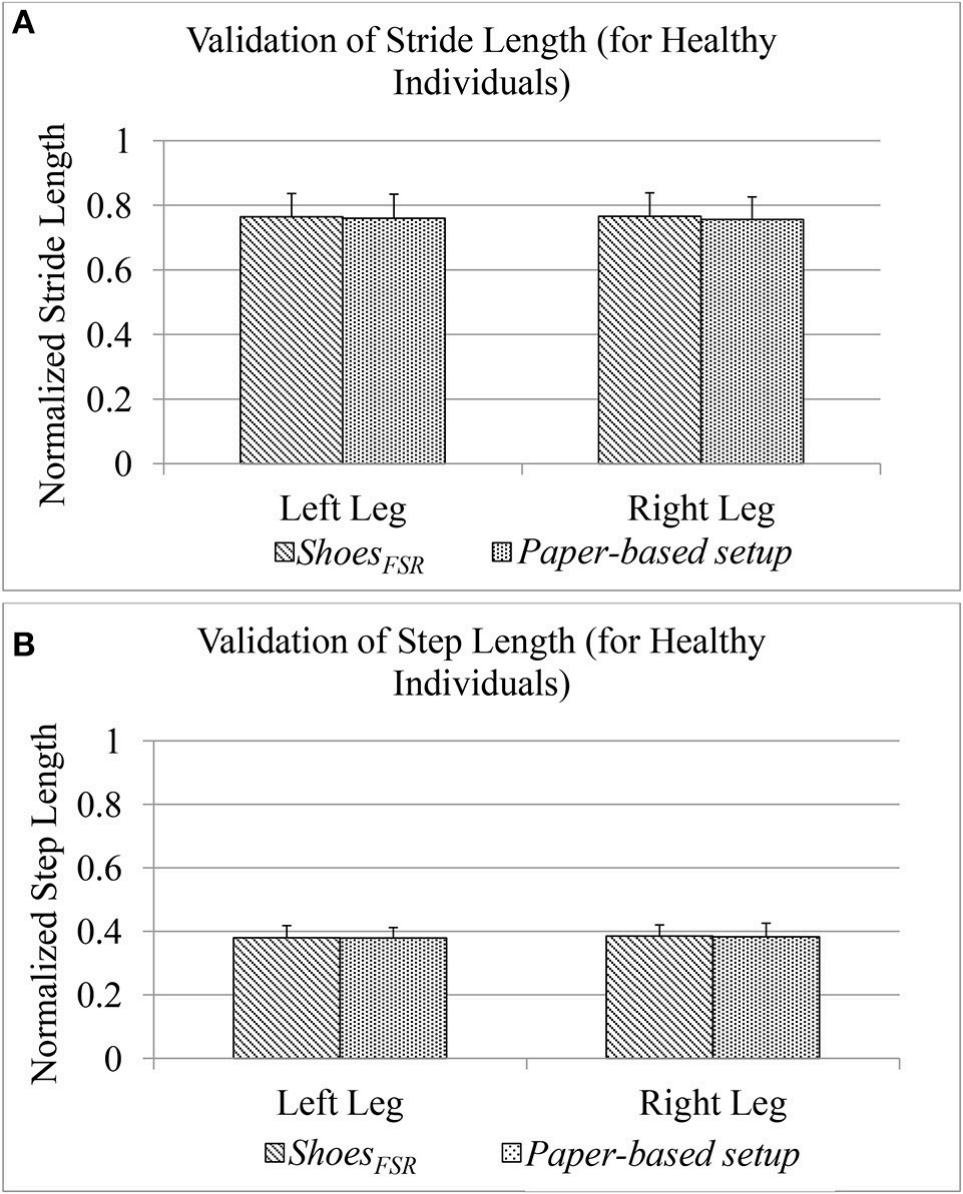
**(A)** Comparison of Stride Length measured using *Shoes*_*FSR*_ and paper-based setup for healthy participants; **(B)** Comparison of Step Length measured using *Shoes*_*FSR*_ and paper-based setup for healthy participants.

Figure 7 shows comparative analysis of *GF*_2_ and *GF*_4_ measured using the *Shoes*_*FSR*_ and the paper-based setup for each leg (Leg_L_ and Leg_R_). The value of % absolute error between the measurements obtained using the *Shoes*_*FSR*_ and the paper-based setup was found to be 0.59 and 1.24% for *GF*_2_; 0.15% and 0.55% for *GF*_4_ for Leg_L_ and Leg_R_, respectively. A good agreement between the measurements done by the two systems is evident from the ICC values > 0.95 for all the cases.

As stated earlier, since our participants of the Stage 2 of our study were healthy individuals, we could observe a close agreement of the spatial gait parameters between Leg_L_ and Leg_R_. Specifically, the difference (%Δ) in the group average of *GF*_2_ and *GF*_4_ between the Leg_L_ and Leg_R_ as measured by *Shoes*_*FSR*_, was ~0.15 and 1.35%, respectively. Similar to the temporal gait parameters, for the spatial gait parameters, we did not find any statistically significant difference (*p* >0.05) between those of Leg_L_ and Leg_R_.

### Understanding the impact of stroke on one's gait

So far we have been validating the parameters measured by our *Shoes*_*FSR*_ with those measured by the standard setups, namely VICON and paper-based setup for healthy participants. Subsequently, we wanted to extend the usage of the *Shoes*_*FSR*_ among the post-stroke survivors. For this, we recruited post-stroke hemiplegic survivors during Stage 3 of the study. In this stage, we wanted to (i) validate the gait parameters measured by the *Shoes*_*FSR*_ with those measured by the paper-based setup followed by a comparison of the gait parameters measured by the two systems and (ii) investigate the feasibility of *Shoes*_*FSR*_ to quantify abnormalities in the gait pattern of post-stroke patients.

#### Results on validation of gait parameters of post-stroke patients measured by *shoes_*FSR*_*

**Figure 8:**
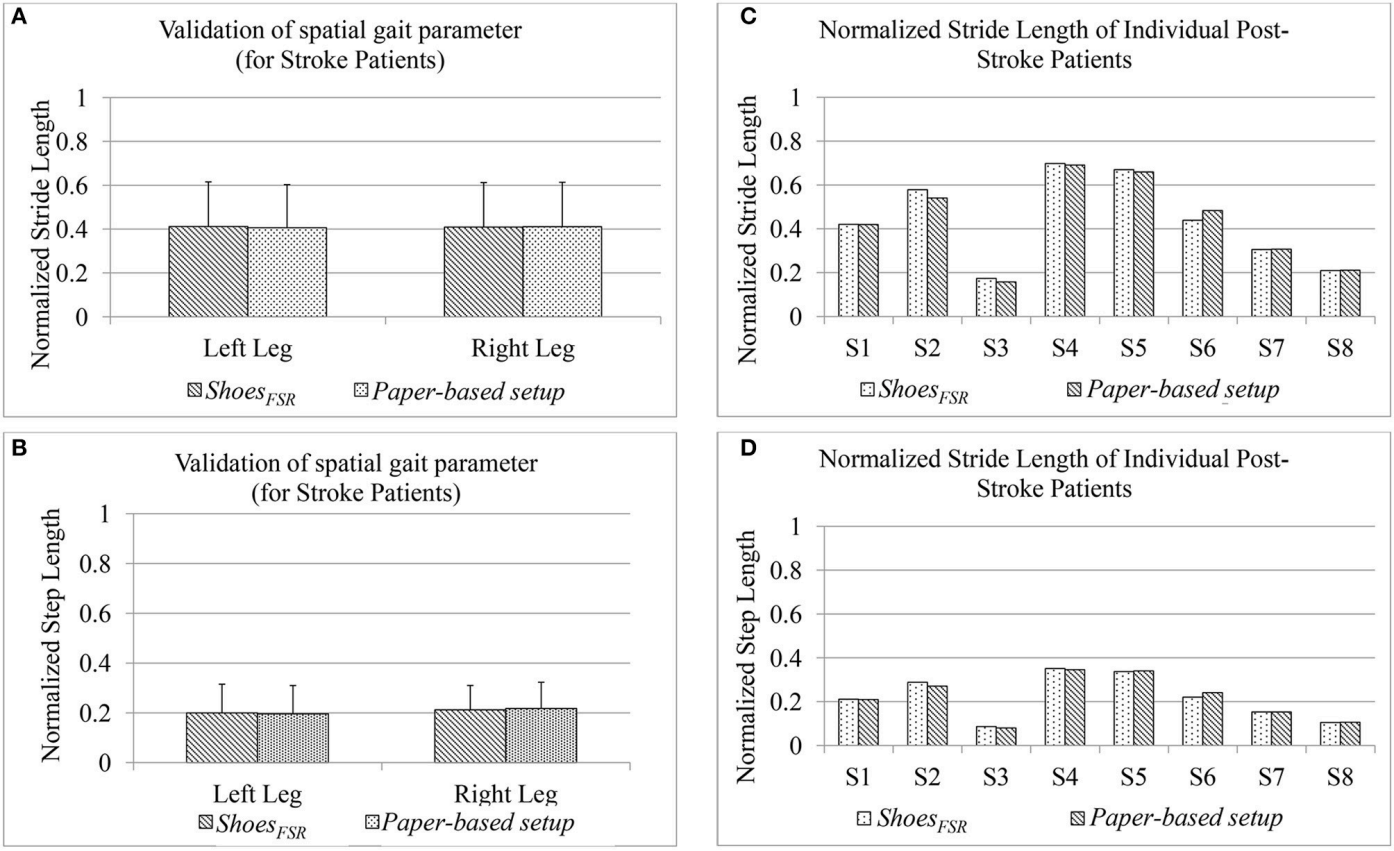
Comparison of normalized **(A)** Stride Length and **(B)** Step Length measured using *Shoes*_*FSR*_ and paper-based setup for post-stroke hemiplegic patients **(C)** Normalized Stride Length of individual post-stroke patients **(D)** Normalized Step Length of individual post-stroke patients.

In Stage 3 of the study, post-stroke hemiplegic participants (S1–S9; Table 2) performed over-ground walk while using (i) *Shoes*_*FSR*_ and (ii) paper-based setup. Figure 8 shows a comparative analysis of some of the gait parameters, e.g., normalized Stride Length (*GF*_2_) and normalized Step Length (*GF*_4_) measured using the *Shoes*_*FSR*_ and the paper-based setup. On an average, the stroke patients showed reduced Stride Length and Step Length as compared to that for the healthy participants (Figures 7A,B) that is in conformity with the reports from literature (O'Sullivan et al., 2013). Also, though the post-stroke patients were hemiplegic, yet, the group average of *GF*_2_ was almost similar across both the legs (Figure 8A). Similar observation was found for *GF*_4_ as well (Figure 8B). This similarity in the group data can be possibly attributed to the fact that nearly 50% of the post-stroke group was right hemiplegic and the rest were left hemiplegic. The group average (excluding S9) of gait parameters measured by *Shoes*_*FSR*_ and the paper-based setup closely matched with % absolute error being 1.58 and 0.15%, respectively for *GF*_2_; and 0.82 and 1.48%, respectively for *GF*_4_ as far as the Leg_L_ and Leg_R_ were concerned. We excluded S9 since his foot imprints were not properly captured on the paper-based setup. Both the measured *GF*_2_ and *GF*_4_ for each leg of the post-stroke group showed larger variation (Figures 8A,B) compared to that for the healthy group (Figures 7A,B). The comparatively larger variation in *GF*_2_ and *GF*_4_ for the post-stroke individuals can be possibly attributed to the spectrum nature of the post-stroke conditions. Since the walking speed varied within hemiplegic patients possessing heterogeneous disability (Table 2), we also present the individual Normalized Stride Length and Step Length as measured by the *Shoes*_*FSR*_ and paper-based setup. As can be seen from Figures 8C,D, irrespective of the individualized capabilities, there existed good agreement between the normalized Stride Length and Step Length values of individual post-stroke patients as measured by *Shoes*_*FSR*_ and paper-based setup. Again, the ICC values confirmed good agreement between the measurements done by the *Shoes*_*FSR*_ and the paper-based setup for post-stroke patients as well (ICC = 0.99 and 0.99, respectively for *GF*_2_; and ICC = 0.98 and 0.93, respectively for *GF*_4_ as far as the Leg_L_ and Leg_R_ were concerned). From these results, we can infer that the *Shoes*_*FSR*_ can be reliably used to quantify gait parameters even for post-stroke hemiplegic individuals. Additionally, using non-parametric test, the statistical analysis revealed a significant difference (*p* < 0.05) for *GF*_4_ but not for *GF*_2_ as far as the paretic and non-paretic legs of post-stroke participants were concerned as indicated in literature (Kirtley, 2006).

#### Implications of stroke on unilateral gait parameters

Till now we have looked into one's gait characteristics using the bilateral gait parameters (such as Step Time, Step Length, Stride Length, etc.) (Buesing et al., 2015). In order to examine the asymmetry in one's gait (especially for hemiplegic post-stroke patients), we analyzed the unilateral gait parameters such as Single Support Time, Swing Time and Stance Time (Von Schroeder et al., 1995).

##### Implication of stroke on single support time (*GF*_5_)

**Figure 9:**
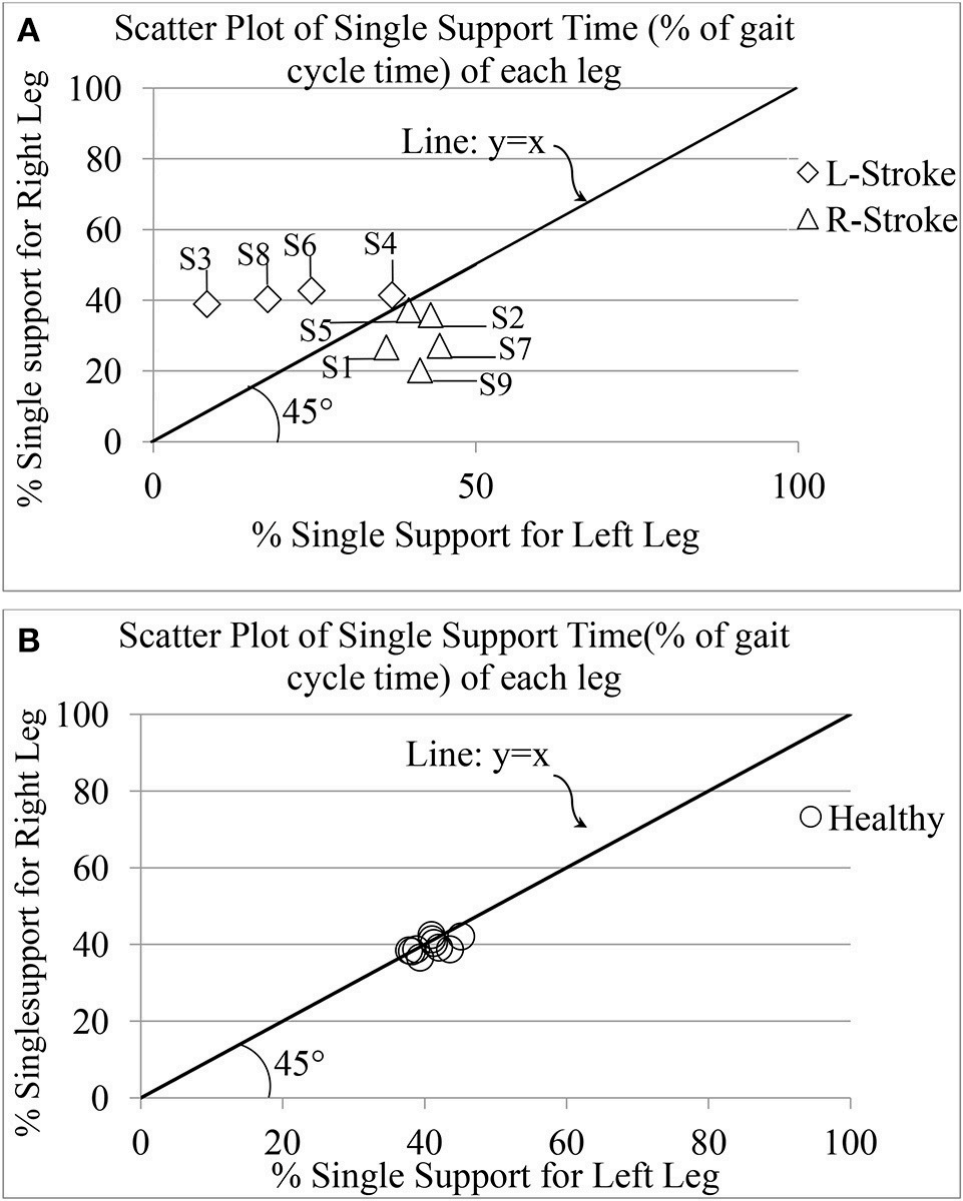
Comparative presentation of single limb support time of each leg for **(A)** post-stroke and **(B)** healthy individuals. R-Stroke, post-stroke patients with right hemiplegia stroke; L-Stroke, post-stroke patients with left hemiplegia.

One's Single (limb) Support Time (*GF*_5_) is an important attribute of one's gait since it has been reported to mirror a post-stroke survivor's functional recovery (Von Schroeder et al., 1995). For healthy gait, one can expect *GF*_5_ associated with one's left (*GF*_5_*Left*_) and right (*GF*_5_*Right*_) legs to be very closely matched. Figure 9 shows the group scatter plot of *GF*_5_*Left*_ vs. *GF*_5_*Right*_ (expressed as % of gait cycle time). From Figure 9, we can observe that for the healthy individuals, the values are clustered close to the line representing y = x, as expected. Again, we can see that for the healthy individuals, the values of *GF*_5_*Left*_ and *GF*_5_*Right*_ were close to 40% that is in agreement with that reported in literature (Kyriazis and Rigas, 2000). In contrast, for all the hemiplegic post-stroke patients (except S2, S4, and S5), these values were distributed on either sides of the line representing y = x in the scatter plot (Figure 9). For S2, S4, and S5, we observed that their corresponding *GF*_5_*Left*_ and *GF*_5_*Right*_ values are in close proximity to the y = x line, similar to that of the healthy participants. A possible explanation for this observation might be that these participants (S2, S4, and S5) demonstrated a low abnormality in their gait compared to the other post-stroke patients, as evident from post-study video analysis. Also, this was supported by the clinical measures as recorded during the 10 m walk test (Table 2).

Again, the post-stroke participant pool comprised of both left hemiplegic (S3, S4, S6, S8) and right hemiplegic (S1, S2, S5, S7, S9; Table 2) patients. In order to understand the implication of the hemiplegic side on the distribution of the values of *GF*_5_*Left*_ and *GF*_5_*Right*_ in the scatter plot, we carried out further analysis. We found that for the left hemiplegic post-stroke patients, *GF*_5_*Left*_<*GF*_5_*Right*_ thereby causing the scatter points to lie to the left of the y = x line. A careful examination of the video analysis revealed that the left hemiplegic patients showed reduced usage of the leg on their paretic side (left leg) that was compensated with increased usage of the leg on their healthy side (right leg) during over-ground walk, similar to that reported in literature (Patterson et al., 2008). In contrast, for the right hemiplegic post-stroke patients, the scatter points were to the right side of the y = x line.

In order to understand whether unilateral gait parameter, such as *GF*_5_ was statistically different as far as the paretic and non-paretic sides of post-stroke hemiplegic participants were concerned, we carried out non-parametric Wilcoxon–Mann–Whitney test. Results indicate that there was statistically significant difference (*p* < 0.05) between the values of *GF*_5_ for the paretic and non-paretic legs.

##### Implication of stroke on swing (*GF*_6_**swing**_) and stance (*GF*_6_**stance**_) phases.

**Figure 10:**
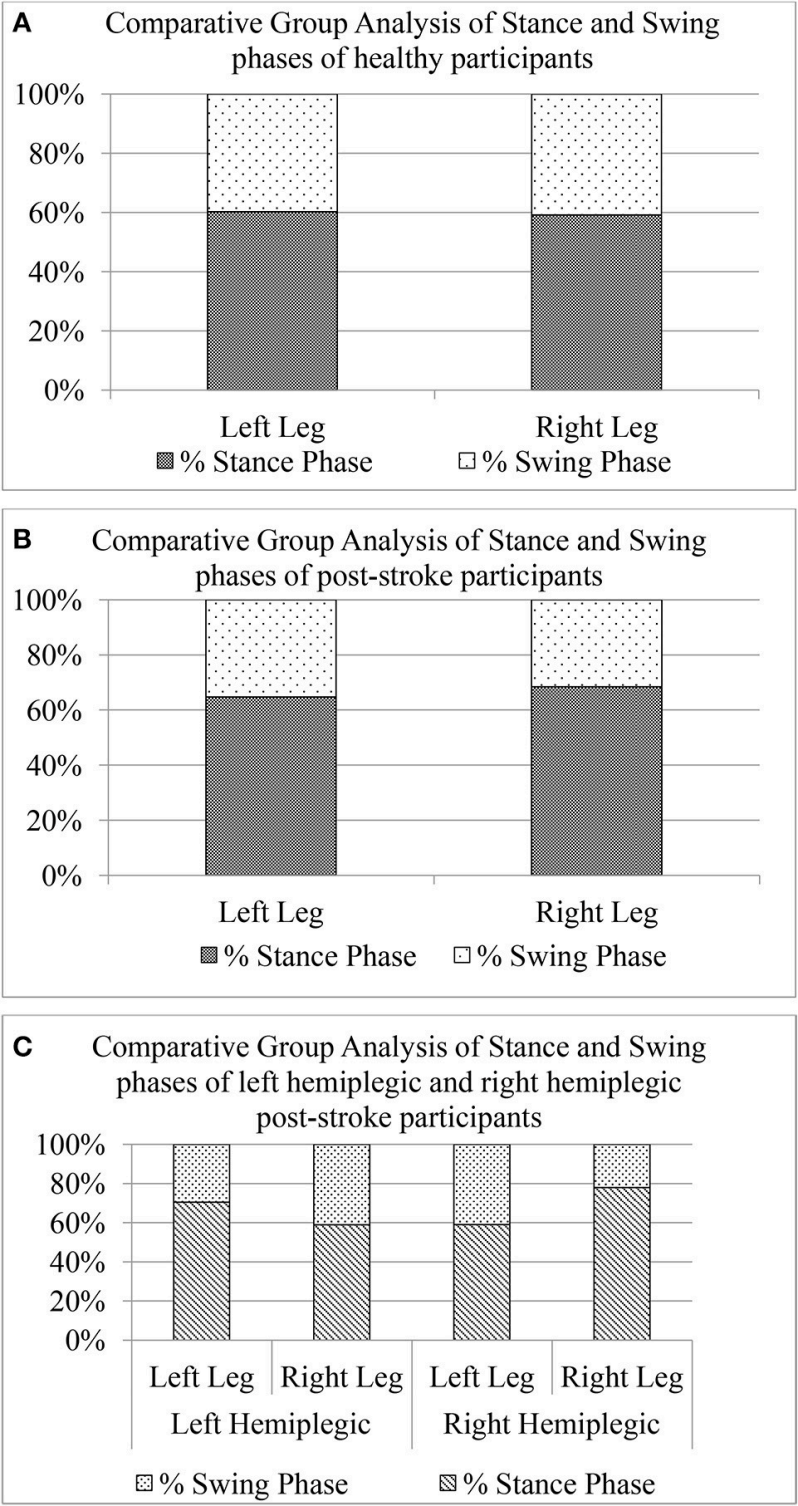
Comparative group analysis of stance and swing stages of left and right legs for **(A)** Healthy participants, **(B)** Post-stroke participants irrespective of hemiplegic side **(C)** Post-stroke participants segregated into left and right hemiplegia.

To further investigate the asymmetry in the gait of hemiplegic post-stroke patients, we calculated the unilateral gait parameters such as Swing and Stance phases quantified as percentage of one's gait cycle time. As reported in literature, healthy gait is characterized by Stance phase being ~60% of gait cycle and Swing phase as the remaining 40% of the gait cycle (O'Sullivan et al., 2013). The Figure 10 shows a comparative group analysis of Stance and Swing phases for healthy and post-stroke participants. It can be seen from Figures 10A,B that for both the healthy and post-stroke participants, the average % Stance and % Swing phases (for Leg_L_ and Leg_R_) were ~60 and 40% of the gait cycle time, respectively. Although the group analysis of post-stroke survivors showed that the % Stance for the Leg_R_ was marginally higher (Δ% = 5.69%) than that for the Leg_L_, the analysis based on hemiplegic side showed a different picture. It can be seen from the Figure 10C that for the right hemiplegic group, the absolute difference in the % Stance between Leg_L_ and Leg_R_ was 12% with that of Leg_L_ being greater of the two. Opposite was the case for the left hemiplegic group in which the absolute difference in the % Stance was 19% with that of Leg_R_ being greater of the two. Also, results on statistical analysis indicated that there was statistically significant difference (*p* < 0.05) between the paretic and non-paretic legs as far as *GF*_6_*Swing*_ and *GF*_6_*Stance*_ were concerned.

##### Implication of stroke on symmetry of weight bearing capacity during one's walk (*GF*_7_)

Understanding symmetry in one's gait is important as it is often related with risk of musculoskeletal injury, particularly in over-used non-paretic limb of post-stroke patients, and challenges in balance during one's walk (Patterson et al., 2008). Thus, in addition to the spatiotemporal data, we tried to understand the asymmetry in weight-bearing capacity of each leg in post-stroke patients by computing symmetry index, i.e., SI (*GF*_7_) using force magnitude data measured by the FSRs (FSR_GD_) in the instrumented shoes (*Shoes*_*FSR*_). Smaller is the value of SI, better is the symmetry and vice-versa for higher value of SI. Results indicate that the asymmetry in weight-bearing capacity of post-stroke group (SI = 25.16 ± 19.07) was higher (%Δ = 68%) compared to that of the healthy group (SI = 14.90± 9.49). This higher value of SI in post-stroke patients can be due to the comparatively reduced usage of the paretic leg than that of the non-paretic leg (Patterson et al., 2008). Thus the *Shoes*_*FSR*_ can offer a precise quantitative estimate of the asymmetry in one's weight-bearing capacity on each leg.

## Discussion and limitation

The main contribution of this study was the design of a cost-effective and portable *Shoes*_*FSR*_ that can characterize one's gait using spatiotemporal gait parameters. The *Shoes*_*FSR*_ consisted of a pair of shoes having FSRs placed under the shoe insole. The FSR output was used to detect gait-related events such as heel strike/off and toe strike/off that were in turn used to measure Stride Length and Time, Step Length and Time, etc. The novelty of the *Shoes*_*FSR*_ was its ability to characterize gait, accommodate cases with foot inversion/eversion, applicability to outdoor use and portability unlike the currently existing techniques. Additionally, this offers a detailed presentation of gait characterization even for post-stroke individuals.

The overall study was conducted in three stages. The Stages 1 and 2 were aimed to explore the ability of *Shoes*_*FSR*_ to measure gait parameters of healthy individuals. In Stage 1, temporal gait parameters measured using *Shoes*_*FSR*_ were validated with that measured using VICON system for healthy individuals. The result showed a good agreement between the measurement of gait parameters done using *Shoes*_*FSR*_ and VICON system. In Stage 2, spatial gait parameters measured using *Shoes*_*FSR*_ were validated with that measured using paper-based setup for healthy individuals. Results validated the reliability of *Shoes*_*FSR*_ to report accurate spatial gait parameters as well. The Stage 3 was aimed to understand the potential of *Shoes*_*FSR*_ to quantify abnormal gait in post-stroke hemiplegic participants. In Stage 3, we validated the gait parameters measured by *Shoes*_*FSR*_ with that measured using the paper-based setup for post-stroke participants. Additionally, we explored the potential of the *Shoes*_*FSR*_ to investigate abnormal gait patterns exhibited by post-stroke patients using different gait-related indices namely, Single Support Time, Swing and Stance phases and Symmetry Index.

Though our results were promising, yet our study had certain limitations. Specifically, one of the limitations was the reduced sample size of post-stroke hemiplegic participants. Also, our participants, enrolled based on availability, had heterogeneous post-stroke conditions. In future, we plan to extend our study by enrolling a larger number of post-stroke participants. Also, to better understand the effect of hemiplegic side on one's gait, we plan to segregate the extended participant pool based on hemiplegic side, age, etc. Additionally, in future, we plan to explore the applicability of *Shoes*_*FSR*_ to different gait patterns exhibited by larger and more diverse group of subjects including healthy subjects simulating different walking patterns as well as post-stroke survivors. Again, with regard to the specifications of the Data Acquisition Module associated with the *Shoes*_*FSR*_, we used a sampling resolution of ~200 samples/second. Though this sampling resolution sufficed for our present application, yet we might need to go for higher sampling resolution to capture gait dynamics for faster gait or while running. Thus, in future, we plan to use hardware with higher sampling resolution when the same *Shoes*_*FSR*_ can be used for other applications as well. Additionally, while we mention that our *Shoes*_*FSR*_ was capable to measure the spatiotemporal parameters of one's gait, yet that was achieved in conjunction with additional devices such as camera. Specifically, as regards the computation of spatial parameters, the *Shoes*_*FSR*_ required information on walking speed for which we have used a camera [section Computation of Stride Length (*GF*_2_)]. However, in future, we plan to use the *Shoes*_*FSR*_ in connection with the Treadmill-based gait rehabilitation with pre-defined speed information that would not need any external device such as camera for separate measurement of walking speed.

However, the results of our preliminary study obtained using *Shoes*_*FSR*_ could distinguish between healthy and pathologic gait and further it could discriminate right hemiplegic and left hemiplegic gait using gait parameters such as % Swing and Stance phases. Also, our results showed the potential of the *Shoes*_*FSR*_ to be used to quantify one's gait symmetry that can help to monitor one's functional gait recovery in a cost-effective manner. At present, we have developed a working prototype of *Shoes*_*FSR*_ that was made in-house with hand-fabricated electronic circuits costing around 90 $. However, we think that the current prototype needs to go through ergonomic modifications that might increase the price of the market-ready version of the *Shoes*_*FSR*_. Presently, the *Shoes*_*FSR*_ have been used in controlled settings such as research labs and hospitals. Thus, questions still remain on the translation of the *Shoes*_*FSR*_ outside the controlled settings to real-world use. Overall the *Shoes*_*FSR*_ showed a commitment to be a reliable, portable and inexpensive solution for characterization of one's gait. In turn, the *Shoes*_*FSR*_ shows a promise for future clinical use.

## Ethics statement

The study was carried out in accordance with the recommendations of Institutional Research Ethics by Institutional Ethics Committee (IEC), IIT Gandhinagar. The protocol was approved by the IEC. All participants provided informed and written consent for their participation in the study.

## Author contributions

DS and UL drafted the manuscript and contributed to the experiment design, experimental data collection with stroke participants, data analysis, and statistical analysis. Also, they read, corrected/commented, and approved the final manuscript.

### Conflict of interest statement

The authors declare that the research was conducted in the absence of any commercial or financial relationships that could be construed as a potential conflict of interest.

## Footnotes

^1^VICON Motion Capture System. Available online at: https://www.vicon.com/products/camera-systems/vero.

^2^Strideway System. Available online at: https://www.tekscan.com/products-solutions/systems/strideway-system?tab=applications).

^3^GAITRite. Available online at: https://www.gaitrite.com/.

^4^VICON LOCK+connect, integrate and synchronize. Available online at: https://www.vicon.com/file/vicon/lock-1104017-24499.pdf.
